# RISK FACTORS FOR FINANCIAL FRAUD AND INTERVENTION STRATEGIES IN DIFFERENT CULTURAL SETTINGS

**DOI:** 10.1093/geroni/igae098.1395

**Published:** 2024-12-31

**Authors:** Hong Mi, Peter Lichtenberg

**Affiliations:** Chair; Discussant

## Abstract

This symposium, comprising five papers, explores financial fraud targeting older adults in mainland China and the U.S.A. Four papers delved into risk factors associated with financial fraud victimization, while one paper examined the efficacy of an intervention effort. The first study, leveraging the 2015 national survey of older Chinese, introduced an algorithm based on machine learning techniques to develop a financial fraud risk prediction model for older adults. This model underscored risk factors like lower education levels, advanced age, and loneliness, alongside protective factors such as awareness of rights protection and quality sleep. Drawing on the 2018 CHARLS data, the second paper investigated both the direct and indirect effects of physical functioning, family dynamics, and individual capital on fraud victimization, revealing psychological vulnerability and material wealth as mediating factors. The third study shifted focus to the U.S.A, profiling victims of postal scams. It identified low education and social isolation risk factors, echoing the patterns observed in the Chinese context in the first paper. The fourth paper examined fraud victimization among a distinct group of older individuals residing in the forest regions of northeastern China, highlighting the roles of financial literacy and internet safety in reducing financial fraud risks. The final paper broadens the research scope by evaluating an intervention designed to aid dementia family caregivers in rural Michigan to mitigate financial exploitation risks. The piloted case management model enhanced caregiver competence in addressing financial exploitation. Together, these studies enrich the understanding of financial fraud affecting older adults in both countries.

